# Protective Effect of Coated Benzoic Acid on Intestinal Epithelium in Weaned Pigs upon Enterotoxigenic *Escherichia coli* Challenge

**DOI:** 10.3390/ani14162405

**Published:** 2024-08-19

**Authors:** Jiawen Qi, Bing Yu, Youjun Hu, Yuheng Luo, Ping Zheng, Xiangbing Mao, Jie Yu, Xiaonan Zhao, Taiqian He, Hui Yan, Aimin Wu, Jun He

**Affiliations:** 1 Institute of Animal Nutrition, Sichuan Agricultural University, Chengdu 611130, China; miaowu777@126.com (J.Q.); ybingtian@163.com (B.Y.); luoluo212@126.com (Y.L.); zpind05@163.com (P.Z.); acatmxb2003@163.com (X.M.); yujie@sicau.edu.cn (J.Y.); yan.hui@sicau.edu.cn (H.Y.); wuaimin0608@163.com (A.W.); 2 Key Laboratory of Animal Disease-Resistant Nutrition, Chengdu 611130, China; 3 Nuacid Nutrition Co., Ltd., Qingyuan 511500, China; 61368851@163.com (Y.H.); zxn780213675@126.com (X.Z.); heqiantai2009@126.com (T.H.)

**Keywords:** coated benzoic acid, enterotoxigenic *Escherichia coli*, intestinal epithelium, weaned pigs

## Abstract

**Simple Summary:**

The research was carried out to examine the safeguarding impact of dietary coated benzoic acid supplementation on intestinal barrier functions in weaned pigs challenged by enterotoxigenic *Escherichia coli*. We found that coated benzoic acid supplementation enhanced the distribution of tight junction proteins, reduced cell apoptosis and intestinal inflammation, as well as increasing immunity and antioxidant capacity in both serum and intestinal epithelium. These results suggest that coated benzoic acid can reduce the damage from enterotoxigenic *Escherichia coli* to intestinal epithelium by reducing inflammation, enhancing intestinal immunity and antioxidant capacity, so as to improve intestinal epithelial function.

**Abstract:**

The study was designed to investigate the protective effect of dietary supplementation with coated benzoic acid (CBA) on intestinal barrier function in weaned pigs challenged with enterotoxigenic *Escherichia coli* (ETEC). Thirty-two pigs were randomized to four treatments and given either a basal diet or a basal diet supplemented with 3.0 g/kg CBA, followed by oral administration of ETEC or culture medium. The results showed that CBA supplementation increased the average daily weight gain (ADWG) in the ETEC-challenged pigs (*p* < 0.05). CBA also increased the serum activity of total superoxide dismutase (T-SOD) and the total antioxidant capacity (T-AOC), as it decreased the serum concentrations of endotoxin, interleukin-6 (IL-6) and tumor necrosis factor-α (TNF-α) in the ETEC-challenged pigs (*p* < 0.05). Interestingly, the CBA alleviated the ETEC-induced intestinal epithelial injury, as indicated by a reversal of the decrease in D-xylose absorption and a decrease in the serum levels of D-lactate and diamine oxidase (DAO) activity, as well as a decrease in the quantity of apoptotic cells in the jejunal epithelium following ETEC challenge (*p* < 0.05). Moreover, CBA supplementation significantly elevated the mucosal antioxidant capacity and increased the abundance of tight junction protein ZO-1 and the quantity of sIgA-positive cells in the jejunal epithelium (*p* < 0.05). Notably, CBA increased the expression levels of porcine beta defensin 2 (*PBD2*), *PBD3*, and nuclear factor erythroid-2 related factor 2 (*Nrf-2*), while downregulating the expression of toll-like receptor 4 (*TLR4*) in the jejunal mucosa (*p* < 0.05). Moreover, CBA decreased the expression levels of interleukin-1β (*IL-1β*), myeloid differentiation factor 88 (*MyD88*), and nuclear factor-kappa B (*NF-κB*) in the ileal mucosa upon ETEC challenge (*p* < 0.05). These results suggest that CBA may attenuate ETEC-induced damage to the intestinal epithelium, resulting in reduced inflammation, enhanced intestinal immunity and antioxidant capacity, and improved intestinal epithelial function.

## 1. Introduction

The intestinal epithelium consists of a single layer of epithelial cells that separates the mucosal tissues from the lumen environment, and is responsible for nutrient and water absorption. It also serves as the mammals’ first line of defence against external antigens and pathogenic microorganisms [1,2]. In swine production, alterations in diet and the removal of maternal antibody protection at weaning activate local or systemic immune responses, impair immunity, and disrupt gastrointestinal homeostasis, leading to various gastrointestinal diseases [3]. Enterotoxigenic *Escherichia coli* (ETEC) is one of the main bacterial causes of post-weaning diarrhea in pigs, and infection stands as a prominent factor contributing to the high rates of illness and death among recently weaned piglets, which account for more than 40% of economic losses in pig production. Importantly, infection with ETEC not only induces diarrhea, but can also induce excessive generation of reactive oxygen species (ROS) and intestinal epithelial cell apoptosis, leading to damage to the intestinal barrier functions [4,5,6,7,8]. In recent decades, a variety of antibiotics have been used for the prevention of diarrhea and intestinal inflammation due to ETEC. However, prolonged or excessive utilization of antibiotics can result in the development of drug resistance or residues, and therefore developing various alternative options to traditional antibiotics has garnered significant attention in the global research community [9].

Acidifiers have been considered to be one of the most prominent substitutes for antibiotics, as they were reported to play a positive role in improving nutrient digestibility, enhancing immunity, and providing antimicrobial effects [10]. Benzoic acid (BA), an organic acidifier, is a colorless crystalline solid that has been used as a green feed additive for pigs [11]. For instance, BA was found to exhibit antimicrobial activity against various pathogenic bacteria including ETEC [12,13]. Moreover, BA also participated in regulating the humoral immune response, antioxidant capacity, and intestinal health in pigs [14,15,16,17]. However, several studies revealed that adding BA to the diet did not affect the growth performance of pigs [18,19,20]. This is probably due in part to their existing forms, as free BA is rapidly absorbed in the stomach and proximal small intestine [21].

To improve the stability of chemicals or drugs, cold spray (CS) technology has been widely utilized [22]. The absorption rates of chemicals or drugs may be delayed by encapsulating them in a matrix of carbohydrates, lipids, or proteins, allowing for their effects to be exerted in the distal small intestine and hindgut. Previous studies indicated that coated organic acid blends can increase the growth efficiency and nutrient utilization in young, developing, and mature pigs [23,24,25]. In recent decades, the CS technique has been well tested in a number of feed additives [26,27]; nevertheless, there is limited knowledge regarding the impact of the CBA on the development and functionality of the intestinal epithelium in pigs challenged with ETEC.

The objective of this research was to examine the safeguarding impact of dietary CBA supplementation on the functions of the intestinal epithelium in weaned pigs that had been exposed to ETEC infection. Our findings indicate that CBA was able to attenuate ETEC-induced diarrhea and the intestinal epithelium disruption associated with a higher abundance of tight junction proteins, to suppress intestinal inflammation and cell apoptosis, and improve intestinal immunity and antioxidant capacity. The positive impact of CBA will also be convincingly indicated by our research, and the potential mechanisms of action were also investigated.

## 2. Materials and Methods

### 2.1. Ethical Approval

The experimental procedures in the present investigation underwent evaluation and authorization by the Animal Experimental Committee of Sichuan Agricultural University (authorization number SICAU-2022-014). All experimental procedures were conducted following the guidelines for the Care and Use of Laboratory Animals.

### 2.2. Animal Diets and Experimental Design

Thirty-two healthy male castrated (Duroc × Landrace × Yorkshire) pigs, weaned at 21 days of age and with an average body weight (BW) of 7.84 ± 0.14 kg, were housed individually in a metabolic cage (0.7 m × 1.5 m × 1.5 m) and allowed to acclimatize to the study conditions for 3 days. The pigs were randomly allocated to a 2 (CBA) × 2 (ETEC) design, resulting in four treatment groups: CON (the basic diet), CBA (3 g/kg CBA), ECON (the basic diet challenged with ETEC), and ECBA (3 g/kg CBA challenged with ETEC). Design of experiment see Table 1 below. CBA was obtained from Guangzhou Nuacid Co., Ltd. (Guangzhou, China). The concentration of CBA at 3 g/kg in the diet is safe for weaned piglets, as approved by the European Union [28]. The experiment continued for 21 days. On day 19, pigs belonging to the ECON and ECBA groups received an oral administration of 200 mL of LB culture, which contained around 2.5 × 10^9^ cfu/mL ETEC K88 (hereafter referred to as ETEC; serotype O149: K91: K88ac, China Veterinary Culture Collection Center, Beijing, China) using an orogastric tube, while pigs in the CON and CBA groups were administered equivalent amount of sterile LB medium. Pigs were located in a temperature- (27 ± 1 °C) and relative-humidity-controlled (65 ± 5%) room and had unlimited access to food and water. Based on the treatment provided, their diets differed. The corn–soybean basal diets (Table S2) were formulated to meet the nutrient requirements for pigs as recommended by the National Research Council [29].

### 2.3. Sample Collection

Blood specimens were collected at 8:00 a.m. on day 22 through jugular vein puncture and placed into two 10 mL nonheparinized vacuum tubes. The serum was prepared by centrifugation of blood samples at 3500× *g* for 15 min at 4 °C and then stored in a refrigerator at −20 °C until analysis. Subsequently, pigs were sacrificed with sodium pentobarbital (200 mg/kg BW). Approximately 4 cm segments from the middle of jejunum duodenum, jejunum and ileum were quickly isolated, washed with cold phosphate buffered saline (PBS), and fixed in PBS for flow cytometry or immunofluorescence and immunohistochemical analysis in 4% paraformaldehyde solution. The remaining segments of the duodenum, jejunum and ileum were opened longitudinally, washed with ice-cold PBS and mucosa samples were obtained by gently scraping with a sterile glass microscope slide at 4 °C. The mucosa samples were quickly frozen in liquid N_2_ and stored at −80 °C refrigerator until further analysis related to antioxidant analysis.

### 2.4. Growth Performance Evaluation

The BW of each pig was monitored at the start and end of the trial, while the consumption of feed and waste feed were recorded and weighed daily. The aforementioned values were utilized in the computation of average daily weight gain (ADWG), average daily feed intake (ADFI), and feed efficiency (F/G). Daily records were kept for the number of pigs experiencing diarrhea. The formula used to calculate the diarrhea rate was as follows: diarrhea rate (%) = (number of pigs with diarrhea within a treatment × total observational days)/(number of pigs × total observational days) × 100% [30]. The incidence of diarrhea was defined by the following standards: fecal score of 0 (normal); fecal score of 1 (normal feces); fecal score of 2 (moist feces), fecal score of 3 (mild diarrhea), fecal score of 4 (severe diarrhea), and fecal score of 5 (watery diarrhea) in all the experiments [31]. Diarrhea was defined as having a stool score of 2 or 3 for two consecutive days.

### 2.5. Serum Parameter Measurements

The levels of tumor necrosis factor-α (TNF-α), interleukin-6 (IL-6), interleukin-1 beta (IL-1β), endotoxin (ET), D-lactate, and the activities of diamine oxidase (DAO) in the serum of the pigs were measured using enzyme-linked immunosorbent (ELISA) assay kits according to the manufacturer’s instructions (Shanghai Meimian Biotechnology Co., Ltd., Shanghai, China).

Malondialdehyde (MDA) levels, total antioxidant capacity (T-AOC) and catalase (CAT), total superoxide dismutase (T-SOD), glutathione peroxidase (GSH-Px) activities were measured in serum using appropriate diagnostic kits (Nanjing Jiancheng Institute of Bioengineering, Jiangsu, China).

### 2.6. Analysis of Intestinal Antioxidant Parameters

Approximately 100 mg of each thawed intestinal mucosa sample extracted from the duodenum, jejunum, and ileum was homogenized by being exposed to precooled 0.9% saline; the sample was centrifuged at 3000× *g*, 4 °C for 15 min to obtain the supernatant. MDA, T-AOC, GSH-Px, T-SOD, and CAT were determined using specific assay kits (Nanjing Jiancheng Institute of Bioengineering, Nanjing, China).

### 2.7. Immunofluorescence Analysis

The jejunal tissue samples were first fixed in 4% paraformaldehyde. Subsequently, they were immersed in xylene I, xylene II, and xylene III for 15 min each. Following this, the samples were placed in anhydrous ethanol for 5 min, anhydrous ethanol II for 5 min, 85% ethanol for 5 min, and 75% ethanol for 5 min. Finally, the samples were washed with distilled water to complete the deparaffinization process. The sections were submerged in citrate buffer with a pH of 6.0, subjected to high microwave power for 10 min, paused for 8 min, and then exposed to medium-high heat for another 10 min. Following the cooling process, the sections underwent three 5 min washes in PBS. Prior to incubation with rabbit anti-ZO-1 polyclonal antibody (1:100; NOVUS), tissue sections were blocked with goat serum albumin overnight at 4 °C. Slides were subjected to three 5 min washes with PBS and then exposed to goat anti-rabbit IgG-FITC secondary antibody (1:100; Servicebio) at 37 °C for 30 min in the dark. Following three rounds of rinsing in PBS for 5 min each, the slides underwent staining with 4′-6-diamidino-2-phenylindole (DAPI, Zhongshan Golden Bridge Biological Co., Ltd., Beijing, China). Nuclei were prepared for identification by incubation for 10 min at room temperature in the dark. The excess DAPI were washed away in PBS 3 times for 5 min. Then the slides were sealed with an anti-fluorescent attenuation sealer.

The zonula occludens 1 (ZO-1) immunofluorescence images were captured using the digital scanning and browsing software version OlyVIA (OLYMPUS, Tokyo, Japan) laser scanning confocal microscope. All sections were first observed at 100×, and 200× microscopic images were acquired, with 3 fields of view in each section. DAPI-stained nuclei are blue and ZO1 shows positive expression in green. The fluorescence intensity (Integrated Density, IntDen) and area (area) of all the acquired high magnification images were determined using Image-J v. 2 (National Institutes of Health, Stapleton, NY, USA) image analysis system, and the mean fluorescence intensity (mean grey value, mean) of each image was calculated, and the mean fluorescence intensity of three images was used to calculate the average, which resulted in the mean fluorescence intensity of the samples in each case.

### 2.8. Cell Cycle Measurement

Jejunal epithelial tissue was rinsed clean in a 60 mm glass dish containing pre-cooled 2 mL PBS, delicately sliced into small fragments using ophthalmic scissors, then softly ground with a grinder; the cell suspension was collected and filtered through a 200-mesh cell sieve. After being centrifuged at 300× *g* for 5 min, the supernatant was removed, the tissue underwent two washes with PBS, then centrifuged again at 300× *g* for 5 min, with the supernatant being discarded once more. Following this, three times the volume of erythrocyte lysis solution was introduced, and the tissue was lysed at room temperature for 5 min before being washed twice with PBS, and centrifuged to collect cellular precipitates for spare use.

Jejunal epithelial cell suspension samples were fixed at −20 °C overnight, the supernatant was discarded after centrifugation at 300× *g* for 5 min, washed twice with the appropriate amount of PBS, centrifuged at 300× *g* for 5 min, the supernatant was discarded, and the cell precipitate was obtained. A measured sample of 500 μL of PI/RNase A staining workup was prepared as the working solution for staining, by the volume of Rnase A: PI according to the volume of 1:9 (ready to be prepared and used now), and cells were suspended in 500 μL of PI/RNase A staining solution, and then detected by CytoFLEX flow cytometry (Beckman Coulter, Inc., Brea, CA, USA) after being kept out of the sunlight for 30 min at room temperature and analyzed using ModFit LT 5.0 software (Verity Software House, Topsham, ME, USA).

### 2.9. Apoptosis Measurement

Jejunal epithelial cells were separated in order to determine the percentage of cells undergoing apoptosis using flow cytometry with a PE Annexin V Apoptosis Detection Kit I (Becton, Dickinson and Company, BD Biosciences, San Jose, CA, USA). Cell suspension specimens underwent centrifugation at 250× *g* for 5 min, followed by discarding the supernatant, two washes with PBS, another centrifugation at 250× *g* for 5 min, and finally discarding the supernatant to acquire the cell precipitate. After suspending the cells in 500 μL of Binding Buffer, 5 μL of Annexin V was gently pipetted into the sample, followed by 5 μL of PI; samples were incubated for 10 min at room temperature in the dark, and subsequently analyzed using flow cytometry (CytoFlex, Beckman Coulter, Inc., Brea, CA, USA).

### 2.10. Immunohistochemistry Analysis of Mucosal sIgA

The abundance of secretory immunoglobulin A (sIgA) was determined in the jejunal epithelium; the 4% paraformaldehyde-fixed jejunal samples were embedded in paraffin wax blocks and sectioned at 2 μm thickness and collected on glass slides. The samples underwent a series of immersion steps, starting with xylene I for 15 min, followed by xylene II for 15 min, xylene III for 15 min, anhydrous ethanol I for 5 min, anhydrous ethanol II for 5 min, 85% alcohol for 5 min, 75% alcohol for 5 min, and finally washing in distilled water to remove paraffin and rehydrate. Subsequently, the sections were placed in citrate buffer (PH 6.0) and microwaved on high for 10 min, followed by an 8 min pause, and then heated again on medium-high for 10 min. After cooling, the sections were washed three times in PBS for 5 min each to repair antigens. Following this, the sections were treated with 3% H_2_O_2_ in methanol for 10 min at room temperature, followed by three washes in PBS for 5 min each time to quench endogenous peroxidase activity. To eliminate non-specific antibody binding, the sections were blocked with 10% goat serum for 20 min at room temperature. Subsequently, overnight incubation at 4 °C was carried out with a 1:200 dilution of goat anti-pig sIgA antibody (Beijing Biosynthesis Biotechnology Co., Ltd., Beijing, China). After washing the sections with PBS thrice for 5 min each, the sections were exposed to biotinylated goat anti-rabbit IgG secondary antibody (Beijing Zhongshan Golden Bridge Biotechnology Co., Ltd., Beijing, China) at 37 °C for 30 min. Subsequently, the sections were washed with PBS thrice for 5 min each time, fresh DAB chromogenic solution was prepared, drops were added to the tissue, the color was developed at room temperature. Color development time can be managed and monitored under the microscope, where a positive result will appear as brownish yellow. Afterwards, the section should be washed with distilled water to terminate the color development. The sections were counterstained with hematoxylin for 3 min, washed with tap water, and rinsed with running water after returning to blue water. Subsequently, the segments underwent dehydration and drying using alcohol of varying concentrations before being sealed with neutral gum following the transparency of xylene. Images of the sections were captured using a microcamera system, and each section was first viewed at low magnification for all tissues, and then three 200× microscopic images were captured. Hematoxylin staining resulted in blue-colored nuclei, and sIgA displayed positive expression in a brownish-yellow shade. The Halo data analysis system was utilized to compute the proportion of positive area in every image (% DAB Positive Tissue).

### 2.11. RNA Extraction, cDNA Synthesis, and Quantitative Real-Time PCR Analysis

Total RNA was extracted from 0.1 g of each frozen sample obtained from the duodenum, jejunum, and ileum using 1 mL of RNAiso Plus reagent (TaKaRa, Dalian, China) following the manufacturer’s guidelines. RNA quality and quantity were rapidly assessed using a spectrophotometer (NanoDrop-ND2000, ThermoFisher Scientific, Inc., Waltham, MA, USA), of which the OD260/OD280 ratio between 1.8 and 2.0 was considered appropriate. Then 1.0 μg of total RNA was utilized for reverse transcription to generate complementary DNA (cDNA) for RT-PCR analysis. The PrimeScript™ RT reagent kit with gDNA Eraser from Takara Biotechnology Co., Ltd. in Dalian, China was employed for this purpose. The quantitative polymerase chain reaction (qPCR) was conducted using the SYBR^®^ Green PCR I PCR reagents (Takara Bio Inc., Dalian, China) on a CFX96 Real-Time PCR Detection System (BioRad Laboratories, Hercules, CA, USA). Each cDNA sample was analyzed in triplicate. For each qPCR reaction (10 μL), 5 µL of SYBR Premix Ex Taq II (Tli RNaseH Plus), 0.4 µL of forward and reverse primers, 1 µL of cDNA, 0.2 μL of 50 × ROX Reference Dye*3, and 3 µL of RNase-Free ddH_2_O were used. The qPCR protocol involved an initial denaturation step at 95 °C for 25 s, followed by 40 cycles of denaturation at 95 °C for 5 s and annealing/extension at 64.5 °C for 25 s. The specificity of the gene amplification products was confirmed through melting curve analysis following each real-time quantitative PCR assay. The 2^−ΔΔCt^ method with β-actin as an internal reference was used to analyze the RNA expression levels of the target genes [32]. All primers in use are in Table S1.

### 2.12. Statistical Analysis

Data were subjected to analysis through a two-way ANOVA utilizing the general linear model (GLM) procedure of SPSS in the form of a 2 (CBA) × 2 (ETEC) factorial design. A *p*-value less than 0.05 was deemed statistically significant, *p*-value in range between 0.05 and 0.1 was treated as tendency. Normality and variance homogeneity assumptions were confirmed using Shapiro–Wilk’s and Levene’s tests, respectively. Duncan’s multiple range test analysis with superscript letters was conducted for one-way ANOVA, revealing a notable distinction. The statistical analysis was carried out using SPSS 27.0 (IBM, Chicago, IL, USA) and GraphPad (version 9) software (GraphPad Software Inc., Solana Beach, CA, USA). The results are presented as means with standard error of a mean for all replications.

## 3. Results

### 3.1. Effect of CBA on Growth Performance in the ETEC-Challenged Pigs

As shown in Table 2, dietary CBA supplementation significantly increased the ADWG and the final body weight in the ETEC-challenged pigs (*p* < 0.05). Compared to the CON group, the ETEC challenge significantly increased the ratio of F:G and the diarrhea ratio (*p* < 0.05). However, dietary CBA supplementation tended to decrease the F:G and diarrhea ratio (0.05 ≤ *p* < 0.10).

### 3.2. Effect of CBA on Serum Inflammatory Cytokines and Antioxidant Capacity in the ETEC-Challenged Pigs

As shown in Table 3, the ETEC challenge significantly increased the serum concentration of IL-1β, but dietary CBA supplementation significantly decreased the IL-1β concentration in the ETEC-challenged pigs (*p* < 0.05). Dietary CBA supplementation also resulted in a decline in the serum concentration of IL-6, TNF-α, and endotoxin (ET) in the ETEC-challenged pigs (*p* < 0.05). The ETEC challenge reduced the activity of CAT, GSH-Px, and T-SOD in serum; however, CBA supplementation significantly elevated their activity in the serum (*p* < 0.05). Dietary CBA supplementation also increased the serum activity of T-AOC, but significantly decreased the serum content of MDA in the ETEC-challenged pigs (*p* < 0.05).

### 3.3. Effect of CBA on Integrity of Intestinal Epithelium in the ETEC-Challenged Pigs

**Figure 1:**
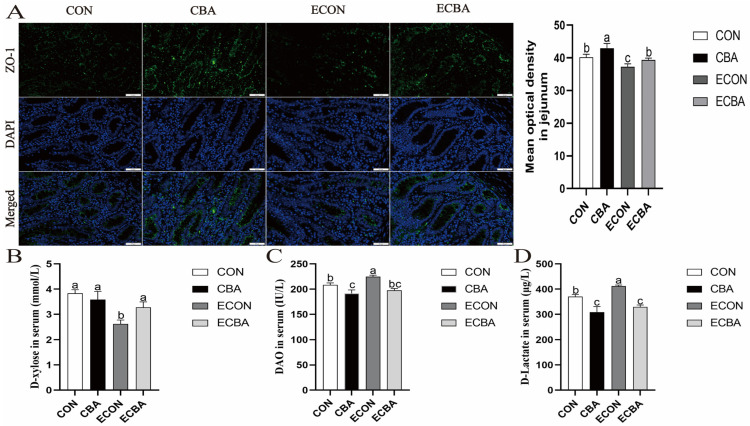
(**A**) Effect of CBA on ZO-1 and DAPI (DNA) distribution in the ETEC-challenged pigs. ZO-1, Zonula occludens 1; DAPI, 4’-6-diamidino-2-phenylindole. ZO-1 protein (green), DAPI stain (blue), and merged ZO-1 protein and DAPI are shown (immunofluorescence; 200×). (**B–D**) Effect of CBA on maintaining intestinal integrity in the ETEC-challenged pigs. DAO, Diamine oxidase. a, b, c mean values within a row with unlike superscript letters were significantly different (*p* < 0.05). CON, pigs were fed with a basal diet; CBA, pigs were fed with a CBA-containing diet, 3 g/kg; ECON, pigs were fed with a basal diet and challenged by ETEC; ECBA, pigs were fed with a CBA-containing diet and challenged by ETEC.

Figure 1 illustrates that the ETEC challenge led to a decrease in the levels of tight-junction protein ZO-1 within the jejunal epithelium and decreased the absorption of D-xylose (*p* < 0.05). However, CBA supplementation led to a significant rise on the abundance of ZO-1 both in the non-challenged and ETEC-challenged pigs, as well as an increase in the D-xylose absorption in the ETEC-challenged pigs (*p* < 0.05). Additionally, CBA supplementation resulted in a notable decrease in the serum concentrations of DAO and D-lactate in the ETEC-challenged pigs (*p* < 0.05).

### 3.4. Effect of CBA Supplementation on Cell Cycle and Apoptosis in the Jejunal Epithelium

**Figure 2:**
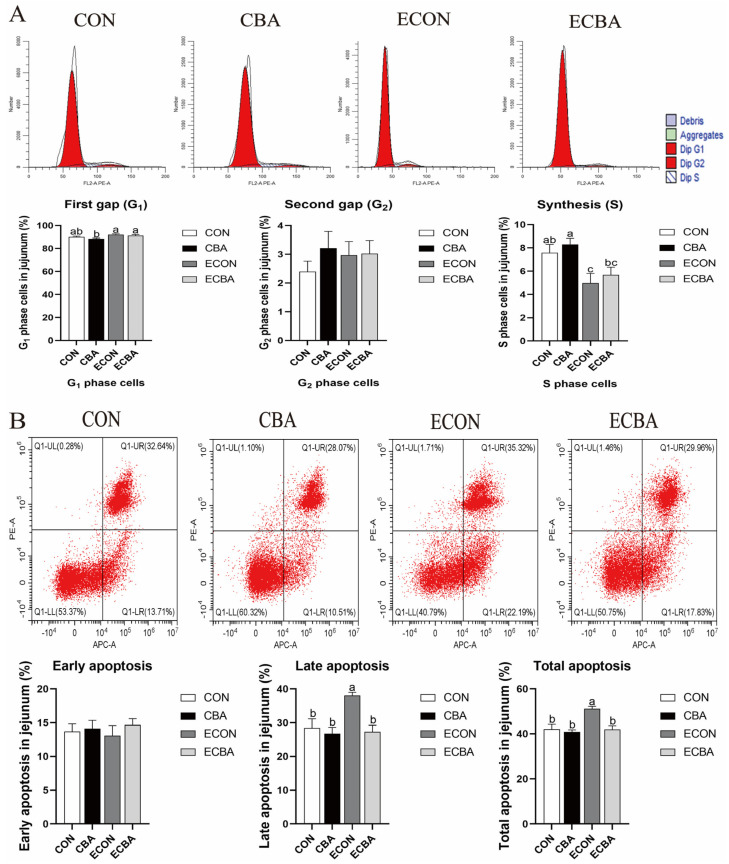
(**A**) Effect of CBA on the jejunal epithelium cell cycle (%) in ETEC-challenged pigs. (**B**) Effect of CBA on the percentage of jejunal epithelium apoptotic cells in ETEC-challenged pigs. A typical example of flow cytometry assays. Q1-UL represents necrotic cells; Q1-UR represents late apoptotic cells; Q1-LL represents normal cells; Q1-LR represents early apoptotic cells. a, b, c mean values within a row with unlike superscript letters were significantly different (*p* < 0.05). CON, pigs were fed with a basal diet; CBA, pigs were fed with a CBA-containing diet, 3 g/kg; ECON, pigs were fed with a basal diet and challenged by ETEC; ECBA, pigs were fed with a CBA-containing diet and challenged by ETEC.

According to Figure 2, the ETEC challenge led to a notable decrease in the quantity of S phase cells within the jejunal epithelium. Conversely, the addition of CBA appeared to have a tendency to elevate the number of S-phase cells in pigs that were challenged with ETEC (0.05 ≤ *p* < 0.10). Moreover, dietary CBA supplementation resulted in a marked decrease in the late and total cell apoptosis rate in the jejunal epithelium upon ETEC challenge (*p* < 0.05).

### 3.5. Effect of CBA on Intestinal Mucosal Immunity and Antioxidant Capacity in ETEC-Challenged Pigs

**Figure 3:**
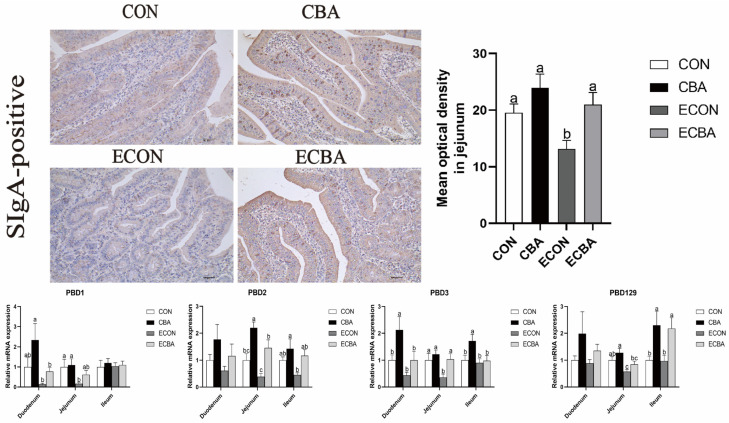
Effects of CBA on the sIgA content and intestinal mucosal antimicrobial peptide genes expression in the jejunum in the ETEC-challenged pigs (immunohistochemistry; 200×). sIgA, secretory immunoglobulin A. a, b, c mean values within a row with unlike superscript letters were significantly different (*p* < 0.05). CON, pigs were fed with a basal diet; CBA, pigs were fed with a CBA-containing diet, 3 g/kg; ECON, pigs were fed with a basal diet and challenged by ETEC; ECBA, pigs were fed with a CBA-containing diet and challenged by ETEC.

As shown in Figure 3, CBA dietary supplementation led to a notable rise in the quantity of sIgA-positive cells in the jejunal epithelium after exposure to ETEC (*p* < 0.05). Dietary CBA supplementation significantly elevated the expression levels of innate immune molecules such as the porcine beta defensin 2 (*PBD2*) and *PBD3* in the jejunal epithelium of the ETEC-challenged pigs (*p* < 0.05). CBA supplementation increased the expression levels of *PBD129* in the ileum both in the non-challenged and ETEC-challenged pigs (*p* < 0.05). As shown in Table 4, dietary CBA supplementation significantly increased the activity of GSH-Px in non-challenged pigs in the duodenum and ileum (*p* < 0.05). ETEC challenge decreased the activity of CAT in the jejunum; however, CBA supplementation tended to increase the activity of GSH-Px in the duodenum upon ETEC challenge (0.05< *p* < 0.10).

### 3.6. Effect of CBA on Expressions of Critical Genes Related to Intestinal Epithelium Functions in ETEC-Challenged Pigs

**Figure 4:**
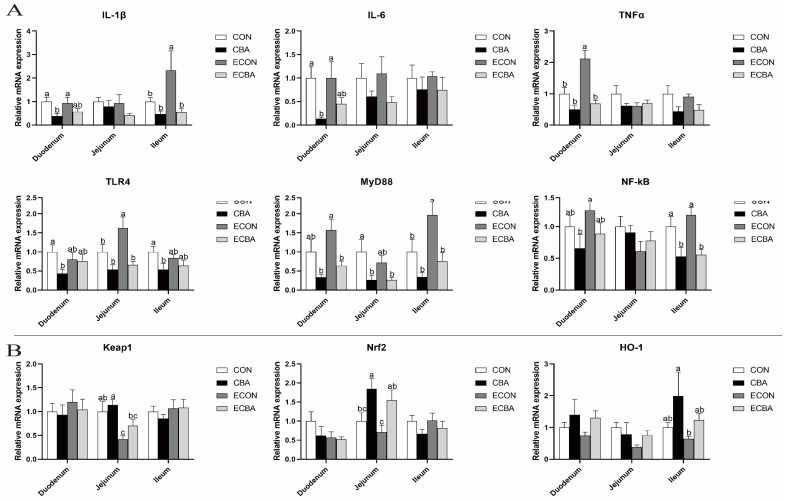
Effect of CBA on intestinal epithelium (**A**) inflammation and (**B**) antioxidant-related gene expressions in ETEC-challenged pigs. *IL-1β*, interleukin-1β; *IL-6*, interleukin-6; *TNF-α*, tumor necrosis factor; *TLR4*, toll-like receptor 4; *MyD88*, myeloid differentiation factor 88; *NF-κB*, nuclear factor-κB; *Keap-1*, Kelch-like ECH-associated protein-1; *Nrf-2*, nuclear factor erythroid 2-related factor 2; *HO-1*, heme oxygenase-1. a, b, c mean values within a row with unlike superscript letters were significantly different (*p* < 0.05). CON, pigs were fed with a basal diet; CBA, pigs were fed with a CBA-containing diet, 3 g/kg; ECON, pigs were fed with a basal diet and challenged by ETEC; ECBA, pigs were fed with a CBA-containing diet and challenged by ETEC.

Figure 4 illustrates that the ETEC challenge resulted in an upregulation of *IL-1β* expression in the ileal mucosa and an increase in *TNF-α* expression in the duodenal mucosa (*p* < 0.05). Nevertheless, dietary CBA supplementation led to a notable reduction in their levels in the pigs exposed to ETEC. Moreover, CBA supplementation also significantly decreased the TLR4 expression in the jejunum, as well as decreased the expression level of *MyD88* and *NF-κB* in the ileum following ETEC challenge (*p* < 0.05). Additionally, CBA supplementation markedly elevated the Nrf2 expression in the jejunum of the ETEC-challenged pigs (*p* < 0.05).

## 4. Discussion

ETEC infection is one of the major causes of bacterial diarrhea in weaned piglets [33], which not only decreases the growth performance [34,35], but also induces intestinal inflammation [36], and impairs intestinal barrier function, leading to increased permeability of the intestine [37,38]. In the present study, we observed that dietary CBA supplementation not only enhanced the ADWG, but also tended to reduce the ratio of F:G and the incidence of diarrhea in the ETEC-challenged pigs. The result is in line with previous reports on pigs using an uncoated BA [39,40,41,42]. The heightened growth efficiency might be a result of better nutrient assimilation and intestinal function [40,43,44].

Serum DAO and D-lactate are two viable and responsive biomarkers present in the circulation, representing intestinal integrity in animals [45]. DAO is normally present in the blood at low levels and is positively correlated with intestinal mucosal integrity [46]. D-lactic is primarily produced by bacteria in the intestine and is not metabolized by mammals [47]. Our results revealed that ETEC challenge significantly enhanced the activity of DAO and the concentration of D-lactate, and decreased the concentration of D-xylose in the serum, which suggested impaired integrity of the intestinal epithelium in the ETEC-challenged pigs. However, CBA supplementation not only reduced the serum activity of DAO and the concentration of D-lactate but also enhanced the concentration of D-xylose in the ETEC-challenged pigs. We analyzed the abundance of ZO-1 protein in the jejunal epithelium to further investigate the protective effects of CBA against ETEC-induced damage to intestinal integrity. As one of the most important proteins of tight junctions, ZO-1 plays a crucial role in intestinal barrier integrity [48,49]. Our study revealed a decrease in the presence of the ZO-1 protein in the jejunal epithelium upon ETEC challenge. Nevertheless, dietary CBA supplementation resulted in an increase in the presence of jejunal ZO-1 protein both in the ETEC-challenged and non-challenged pigs. These results suggested improved intestinal integrity and tight junction protein localization upon CBA supplementation, which may have contributed to the improvement in the growth performance in the ETEC-challenged pigs.

The typical eukaryotic cell cycle is composed of G1, S, G2, and M phases [50]. A previous study showed that ETEC challenge led to perturbation of the jejunal epithelial cell cycles, with a notable G1 arrest, and subsequently caused a decrease in the quantity of cells present in the G2 and S phases [51]. In this study, the ETEC challenge caused G1 arrest in the jejunum, and reduced the number of S-phase cells. However, CBA supplementation tended to increase the number of S-phase cells. Apoptosis is a biological event that regulates cell numbers and removes cells activated by an endogenous signal following exposure to toxins, and also in reaction to disruptions in the cell cycle [52]. Another previous study indicated that ETEC infection significantly increased the percentage of apoptotic epithelial cells in the small intestine, which subsequently contributed to disruption of the intestinal epithelial cells [53]. In our study, CBA supplementation notably reduced the late and total apoptosis rates of jejunal epithelial cells in the ETEC-challenged pigs. This result is also in line with the previous study on mice [54]. Both results demonstrated the advantageous impact of CBA in improving the intestinal epithelium functions in the weaned pigs upon ETEC challenge.

Weaning stress and ETEC infection lead not only to disruption of the intestinal epithelium but also to overproduction of ROS and have been linked in previous studies [55,56]. Previous studies also indicated that BA could reduce ROS levels in the body and prevent oxidative damage [57,58]. In this study, CBA supplementation increased the serum activities of GSH-Px, T-SOD and T-AOC, indicating an improved antioxidant capacity in the pigs. ETEC infection causes an imbalance in the levels of pro-inflammatory and anti-inflammatory cytokines within the body, which leads to stimulation of the mucosal immune system and triggers inflammation and damage to the tissues [59,60]. ET is the major component of the outer membrane of ETEC and has been suggested to activate pro-inflammatory cytokines such as TNF-α, IL-6, and IL-1β which have the potential to induce harm to the structure and operation of the gastrointestinal tract [61,62]. In this study, CBA supplementation led to a notable reduction in the serum levels of IL-1β, IL-6, TNF-α, and ET, as well as a decrease in the expression of pro-inflammatory cytokines *IL-1β* and *TNF-α* in the intestines of weaned pigs following ETEC challenge, indicating a suppressed inflammatory reaction. It is well known that inflammatory responses involve multiple signaling pathways [63], in which TLR4 proactively recognizes ET from ETEC, leading to the secretion of a variety of pro-inflammatory cytokines via the MyD88/NF-κB signaling pathway [64]. The current investigation found that CBA supplementation led to a reduction in the expression levels of *TLR4* and *MyD88* in the duodenal mucosa, as well as *MyD88* and *NF-κB* in the ileal mucosa following the ETEC challenge. This finding is consistent with the assessment of the serum inflammatory cytokines. These findings suggest that ETEC-induced inflammatory response may be inhibited by CBA by obstruction of the MyD88/NF-κB signaling pathway.

SIgA is primarily involved in the intestinal mucosal pathogen-specific immune response and can target polysaccharides and flagellin on the bacterial surface, preventing attachment to the luminal surface and infection [65]. A previous study showed that the jejunal epithelial sIgA concentration decreased in weaned pigs after ETEC challenge. In this study, the ETEC challenge was observed to decrease the presence of sIgA-positive cells in the jejunal epithelium. Conversely, CBA supplementation significantly increased the presence of sIgA-positive cells in pigs subjected to ETEC challenge. As an important component of the specific immune response, antibacterial proteins can act as a critical regulator of the immune response [66]. *PBD1*, *PBD2* and *PBD3* exhibit high expression levels in pig tissues and play crucial roles in the immune system, *PBD129* showed significant antimicrobial activity against ETEC [67,68]. ETEC challenge decreased the jejunal expression level of *PBD1*, *PBD3* and *PBD129*. However, CBA supplementation increased *PBD2* and *PBD3* expression levels in the jejunum and *PBD129* in the ileum upon ETEC challenge. These results are consistent with the abundance of sIgA. Furthermore, the presence of the antioxidant enzyme and its byproduct in the small intestine mucosa was identified. In the non-challenged groups, CBA supplementation increased the activity of GSH-Px in the duodenal and ileum mucosa and reduced the concentration of MDA in the jejunal mucosa. Both results indicate that CBA has the potential to enhance the intestinal antioxidant capacity by increasing the activity of antioxidant enzymes. Nrf2 is a pivotal regulator of the expression of antioxidant enzymes [69]. The Nrf2-Kelch-like ECH-associated protein 1 (Keap1) system plays a pivotal role in regulating the biological response to oxidative stress, which could control the transcription of numerous antioxidant genes that maintain cellular homeostasis [70]. In this study, CBA supplementation resulted in a notable increase in the *Nrf2* expression within the jejunal mucosa, consistent with the improvements in the activities of key antioxidant enzymes. This suggests that CBA exerts a protective effect on intestinal oxidative damage caused by *E. coli* through the Nrf2-Keap1 pathway.

## 5. Conclusions

Our findings suggest that dietary CBA supplementation has a protective impact on weaned pigs when faced with ETEC challenge. This protection may be linked to enhanced distribution of tight junction proteins, reduced cell apoptosis and intestinal inflammation, as well as increased immunity and antioxidant capacity in both serum and intestinal epithelium.

## Figures and Tables

**Table 1:** Design of experiment (n = 32).

Factors		ETEC	
		No Challenge	Challenge with *E. coli*
CBA	No	CON (n = 8)	ECON (n = 8)
	Yes	CBA (n = 8)	ECBA (n = 8)

**Table 2:** Effect of CBA on growth performance in the ETEC-challenged pigs (1-21d).

ITEM	Treatment				*p*-Value (One-Way ANOVA)	SEM (n = 32)	*p*-Value		
	CON	CBA	ECON	ECBA			CBA	ETEC	Interaction
Initial body weight	7.99	7.78	7.66	7.85	0.373	0.07			
Final body weight	13.03 ^a^	13.73 ^a^	11.41 ^b^	13.14 ^a^	0.013	0.27	0.014	0.025	0.279
ADFI (g/d)	403.01 ^ab^	421.15 ^a^	319.13 ^b^	406.77 ^ab^	0.100	15.68	0.085	0.108	0.250
ADWG (g/d)	240.08 ^ab^	283.33 ^a^	178.90 ^b^	251.79 ^a^	0.021	12.34	0.013	0.043	0.503
F: G	1.73 ^ab^	1.51 ^b^	1.84 ^a^	1.65 ^ab^	0.142	0.05	0.045	0.217	0.898
Diarrhea ratios (%)	6.55 ^b^	4.76 ^b^	26.79 ^a^	13.10 ^ab^	0.013	2.77	0.121	0.006	0.229

ADFI, average daily feed intake; ADWG, average daily weight gain; F: G, feed: gain ratio. Data are means of 8 replicates per treatment. a, b mean values within a row with unlike superscript letters were significantly different, *p* < 0.05. CON, pigs were fed with a basal diet; CBA, pigs were fed with a CBA-containing diet, 3 g/kg; ECON, pigs were fed with a basal diet and challenged by ETEC; ECBA, pigs were fed with a CBA-containing diet and challenged by ETEC.

**Table 3:** Effect of CBA on serum inflammatory cytokines and antioxidant capacity in the ETEC-challenged pigs.

ITEM	Treatment				*p*-Value (One-Way ANOVA)	SEM (n = 32)	*p*-Value		
	CON	CBA	ECON	ECBA			CBA	ETEC	Interaction
IL-6 (ng/L)	748.00 ^ab^	693.23 ^bc^	805.50 ^a^	619.01 ^c^	<0.001	17.75	<0.001	0.757	0.020
IL-1β (ng/L)	34.31 ^b^	35.87 ^ab^	39.53 ^a^	34.38 ^b^	0.045	0.77	0.211	0.193	0.024
TNF-α (pg/mL)	270.69 ^a^	259.77 ^a^	263.92 ^a^	205.60 ^b^	<0.001	5.99	<0.001	<0.001	0.006
ET (pg/mL)	300.16 ^ab^	292.17 ^b^	316.37 ^a^	290.59 ^b^	0.069	3.91	0.027	0.322	0.231
CAT (U/mL)	31.10 ^a^	29.06 ^a^	15.87 ^b^	16.70 ^b^	<0.001	1.88	0.838	<0.001	0.633
GSH-Px (μmol/L)	478.04 ^a^	456.53 ^ab^	405.39 ^b^	492.24 ^a^	0.033	11.52	0.127	0.382	0.015
MDA (nmol/mL)	3.02 ^b^	3.17 ^b^	4.01 ^a^	3.58 ^ab^	0.064	0.15	0.613	0.015	0.293
T-AOC (U/mL)	3.36 ^bc^	4.56 ^ab^	2.19 ^c^	6.20 ^a^	0.004	0.44	0.001	0.747	0.066
T-SOD (U/L)	175.83 ^ab^	171.49 ^b^	153.49 ^c^	182.18 ^a^	<0.001	2.41	<0.001	0.070	<0.001

IL-6, interleukin-6; IL-1β, interleukin-1 beta; TNF-α, tumor necrosis factor alpha; ET, endotoxin; CAT, catalase; GSH-Px, glutathione peroxidase; MDA, malondialdehyde; T-AOC, total antioxidant capacity; T-SOD, total superoxide dismutase. Data are means of 8 replicates per treatment. a, b, c mean values within a row with unlike superscript letters were significantly different, *p* < 0.05. CON, pigs were fed with a basal diet; CBA, pigs were fed with a CBA-containing diet, 3 g/kg; ECON, pigs were fed with a basal diet and challenged by ETEC; ECBA, pigs were fed with a CBA-containing diet and challenged by ETEC.

**Table 4:** Effect of CBA on intestinal mucosal antioxidant capacity in ETEC-challenged pigs.

ITEM	Treatments				*p*-Value (One-Way ANOVA)	SEM (n = 32)	*p*-Value		
	CON	CBA	ECON	ECBA			CBA	ETEC	Interaction
Duodenum									
CAT (U/mL)	7.68	10.30	9.81	9.09	0.867	1.12	0.685	0.854	0.478
GSH-Px (μmol/L)	39.17 ^b^	87.46 ^a^	37.54 ^b^	52.60 ^b^	0.010	6.30	0.007	0.105	0.138
MDA (nmol/mL)	0.81	0.86	0.76	0.63	0.730	0.07	0.357	0.776	0.562
T-AOC (U/mL)	0.17	0.24	0.17	0.23	0.808	0.03	0.346	0.938	0.926
T-SOD (U/L)	108.56	122.83	117.41	108.92	0.662	4.63	0.763	0.792	0.240
Jejunum									
CAT (U/mL)	11.74 ^a^	9.40 ^a^	5.87 ^b^	6.20 ^b^	<0.001	0.64	0.315	<0.001	0.184
GSH-Px (μmol/L)	32.29	48.04	30.97	42.63	0.511	4.59	0.151	0.719	0.827
MDA (nmol/mL)	1.84 ^a^	1.24 ^b^	1.45 ^ab^	1.19 ^b^	0.016	0.09	0.009	0.154	0.275
T-AOC (U/mL)	0.11	0.15	0.11	0.12	0.779	0.02	0.482	0.567	0.623
T-SOD (U/L)	156.83	153.66	182.23	176.47	0.295	6.31	0.723	0.063	0.918
Ileum									
CAT (U/mL)	3.27 ^ab^	4.80 ^a^	2.59 ^b^	3.38 ^ab^	0.071	0.31	0.056	0.081	0.529
GSH-Px (μmol/L)	63.61 ^b^	108.85 ^a^	53.48 ^b^	70.12 ^b^	0.001	5.82	0.002	0.014	0.134
MDA (nmol/mL)	0.92	0.64	0.91	0.83	0.409	0.06	0.172	0.518	0.444
T-AOC (U/mL)	0.08	0.11	0.10	0.13	0.416	0.01	0.162	0.380	0.798
T-SOD (U/L)	138.25	122.36	124.04	114.12	0.327	4.61	0.169	0.230	0.747

Data are means of 8 replicates per treatment. a, b, mean values within a row with unlike superscript letters were significantly different, *p* < 0.05. CON, pigs were fed with a basal diet; CBA, pigs were fed with a CBA-containing diet, 3 g/kg; ECON, pigs were fed with a basal diet and challenged by ETEC; ECBA, pigs were fed with a CBA-containing diet and challenged by ETEC.

## Data Availability

The data outlined in the paper, along with the code book and analytical code, can be accessed upon request.

## Supplementary Materials

The following supporting information can be downloaded at: https://www.mdpi.com/article/10.3390/ani14162405/s1, Table S1: Sequences of primers for genes; Table S2: Experiment with basal diet composition and nutrient level.
