# Supplementary material for: Protective Effect of Coated Benzoic Acid on Intestinal Epithelium in Weaned Pigs upon Enterotoxigenic Escherichia coli Challenge
Source: Animals (Basel). 2024 Aug 19;14(16):2405. doi: 10.3390/ani14162405 (PMC11350680; doi:10.3390/ani14162405)
Supplement: Supplementary file 1 [file animals-14-02405-s001.zip › Table s1.pdf]

Table S1. Sequences of primers for genes

Gene	Primer sequence (5' to 3')	Annealing temperature, ℃	Product size, bp
β-Actin	F: TGGAACGGTGAAGGTGACAGC<br>R: GCTTTTGGGAAGGCAGGGACT	60	177
PBD1	F: CTCCTCATGGTCCTGTTACCT<br>R: TGCCGATCTGTTTCATCTTTGG	60	146
PBD2	F: TGTCTGCCTCCTCTCTTCC<br>R: AACAGGTCCCTTCAATCCTG	60	149
PBD3	F: CCTTCTCTTTGCCTTGCTCTT<br>R: GCCACTCACAGAACAGCTACC	60	163
PBD129	F: GCACACTTGAAGAGGTCGCCA<br>R: ATGCTGGCGAAAGGGTTGGT	60	121
IL-1β	F: CAGCTGCAAATCTCTCACCA<br>R: TCTTCATCGGCTTCTCCACT	60	112
IL-6	F: TTCACCTCTCCGGACAAAAC<br>R: TCTGCCAGTACCTCCTTGCT	60	122
TNF-α	F: CGTGAAGCTGAAAGACAACCAG<br>R: GATGGTGTGAGTGAGGAAAACG	60	121
TLR4	F: TCAGTTCTCACCTTCCTCCTG<br>R: GTTCATTCCTCACCCAGTCTTC	60	166
MyD88	F: GATGGTAGCGGTTGTCTCTGAT<br>R: GATGCTGGGGAACTCTTTCTTC	60	148
NF-κB	F: CGTGAAGCTGAAAGACAACCAG<br>R: GATGGTGTGAGTGAGGAAAACG	60	121
Keap1	F: ACGACGTGGAGACAGAAACGT<br>R: GCTTCGCCGATGCTTCA	60	56
Nrf2	F: GCCCCTGGAAGCGTTAAAC<br>R: GGACTGTATCCCCAGAAGGTTGT	60	67
HO-1	F: GCTGAGAATGCCGAGTTCAT	60	142

	R: TGTAGACCGGGTTCTCCTTG

PBD1, porcine beta defensin 1; *PBD2*, porcine beta defensin 2; *PBD3*, porcine beta defensin 3; *PBD129*, porcine beta defensin 129; *IL-1β*, interleukin-1 beta; *IL-6*, interleukin-6; *TNF-α*, tumour necrosis factor-α; *TLR4*, toll-like receptor 4; *MyD88*, myeloid differentiation factor 88; *NF-κB*, nuclear factor-kappa B; *Keap1*, Kelch-like ECH-associated protein-1; *Nrf2*, nuclear factor erythroid-2 related factor 2; *HO-1*, Heme Oxygenase-1.
