# Supplementary material for: Protective Effect of Coated Benzoic Acid on Intestinal Epithelium in Weaned Pigs upon Enterotoxigenic Escherichia coli Challenge
Source: Animals (Basel). 2024 Aug 19;14(16):2405. doi: 10.3390/ani14162405 (PMC11350680; doi:10.3390/ani14162405)
Supplement: Supplementary file 1 [file animals-14-02405-s001.zip › Table s2.pdf]

Table S2. Experiment with basal diet composition and nutrient level

Ingredients	%	Nutrient level	contents
Corn	26.73	Digestible energy, calculated, MJ/kg	3.55
Extruded corn	25.45	Crude Protein, %	19.80
Soybean meal	9.50	Calcium, %	0.92
Extruded full-fat soybean	10.50	Available phosphorus, %	0.37
Fish meal	4.00	Lysine, %	1.41
Whey powder	7.00	Methionine, %	0.47
Soybean protein concentrate	8.00	Methionine + cysteine, %	0.75
Soybean oil	2.00	Threonine, %	0.79
Sucrose	4.00	Tryptophan, %	0.22
Limestone	0.90		
Dicalcium phosphate	0.50		
NaCl	0.30		
L -Lysine HCl, 78%	0.47		
DL-Methionine	0.15		
L -Threonine, 98.5%	0.13		
Tryptophan, 98%	0.03		
Chloride choline	0.10		
Vitamin premix [1]	0.04		
Mineral premix [2]	0.20		
Total	100		

[1]The vitamin premix provided the following per kg of diet: 6000 IU of VA, 400 IU of VD 3, 10 IU of VE, 2 mg of VK 3, 0.8 mg of VB1, 6.4 mg of VB 2, 2.4 mg of VB6, 12 ug of VB12, 14 mg of niacin, 10 mg of pantothenic acid, 0.2 mg of folic acid. [2] The mineral premix provided the following per kg of diet: 120 mg Fe, 6 mg Cu, 100 mg Zn, 40 mg Mn, 0.3 mg I, 0.3 mg Se.

[2]The diet was formulated based on the recommendation of NRC2012.
